# Theory of Mind in ADHD. A Proposal to Improve Working Memory through the Stimulation of the Theory of Mind

**DOI:** 10.3390/ijerph17249286

**Published:** 2020-12-11

**Authors:** Rocío Lavigne, Antonia González-Cuenca, Marta Romero-González, Marta Sánchez

**Affiliations:** 1 Department of Developmental and Educational Psychology, University of Malaga, 29071 Malaga, Spain; rlc@uma.es; 2 Neuropsipe, Child and Adolescent Neuroscience Center, 29010 Malaga, Spain; mromero@neuropsipe.com (M.R.-G.); msanchez@neuropsipe.com (M.S.)

**Keywords:** ToM, WM, VC, ADHD, primary education

## Abstract

The aim of this study was to investigate the relationships between Theory of Mind (ToM), Working Memory (WM), and Verbal Comprehension (VC). Performance of these variables was evaluated in 44 elementary students (6–12 years) diagnosed with ADHD. Their performance in all variables was collected through the Neuropsychological Battery (NEPSY-II) and the Wechsler Intelligence Scale for Children IV. The results showed that fifty percent of the participants were below the 25th percentile in ToM and that this low performance was not related to age. In addition, analyses showed statistically significant relationships between WM, VC, and ToM. Analysis of the effect of WM and VC on ToM showed that only WM explained the variance in participant performance in ToM. These results led us to raise the need to include ToM among the skills to be stimulated in programs for the treatment of ADHD, accompanying other skills related to social adaptation that are usually included in such programs. Likewise, considering that ToM implies putting into practice skills such as considering different points of view, attending to relevant aspects of the context, making decisions, inferring mental states, and predicting behaviors, we believe that through the stimulation of ToM, WM would also be stimulated.

## 1. Introduction

Attention Deficit Hyperactivity Disorder (ADHD) is characterized by a persistent pattern of hyperactivity/impulsivity and/or inattention (higher than expected according to the individual’s developmental level) for at least six months in two or more settings and appearing before the age of twelve, altering the normal functioning of the person in question [1].

It is a severe syndrome caused by a delay in neuropsychological development, which leads to dysfunctions in the mechanisms of Executive Control and Behavior Inhibition. Therefore, the disorder directly affects the Psychological Processes of the Executive System—working memory, attention, self-regulation of motivation and emotions, internalization of language, and processes of analysis and synthesis—and, consequently, executive functions such as planning, organization, decision making, etc. These functions are directly involved in teaching–learning tasks and are necessary for a correct family, academic, and social adaptation [2,3,4,5,6].

These cognitive skills play an essential role in the development and learning of children from 6 to 12 years of age in primary education, since they are directly involved in positive academic and social performance. In this demanding period, students are expected to be able to learn and comply with rules, show resistance to distracting stimuli, be able to plan and organize themselves in advance, solve school and social problems without the supervision of parents and teachers, etc. These self-regulatory activities require good executive control [7].

Compared to control groups, different studies reveal that children with ADHD have difficulties in controlling their behavior, which leads to them failing in school and showing difficulties in interpreting social cues, in their interpersonal skills, and therefore, in their relationships with others, which are often not as satisfactory as they should be. All this means that, on the one hand, they are uninhibited, distracted, absent-minded, forgetful, disorganized, and not very persistent, and, on the other hand, oppositionist, and defiant, with a poorer capacity of adaptation [8] and scarce social skills. In addition, they show difficulties in respecting turns of speech, controlling interruptions, recognizing emotions in themselves and in others, as well as in showing adequate empathic capacity [9,10,11,12,13].

Ultimately, people diagnosed with the disorder have a low degree of self-control and consequently, a limited number of basic skills and inadequate motivation for school and social functioning. Classmates of children with ADHD often complain about the difficulties in maintaining interpersonal relationships with them [5], and children with this disorder often require continuous attention or supervision by the teacher to carry out their functions (for example, following the rules in games). Social relationships are complicated because in addition to presenting intrusive behaviors, these children are not able to comprehend the negative effect of such behaviors on the social environment. Moreover, this type of behavior remains constant over time, increasing its impact during adolescence.

### 1.1. Theory of Mind, Language, and Executive Function

In recent decades, the Theory of Mind (ToM) construct has received enormous attention. This is due to the fact that it is a skill that combines both the cognitive and social competence of individuals, constituting a key facet of social cognition. Originally, ToM was defined as the ability to attribute mental states to oneself and others, as well as to reason about those mental states which are not observable (e.g., beliefs, knowledge, thoughts) to make predictions about one’s own behavior and that of others [14]. Defined in this way, ToM is a cognitively complex acquisition that differs from other simpler social skills such as general sociability, emotion recognition, and empathy. 

Although the assessment of ToM has changed over time, the best known evaluations are classics such as the “change of location” task [15] and the “unexpected content” or “smarties” task [16]. Both require the respondent to predict the behavior of a character based on the attribution of a false belief. There is a broad consensus that between the ages of 5 and 6—initially 4 and a half was marked as a milestone—success in these tasks is widespread. The meta-analysis by Wellman et al. (2001) [17] shows a clear coincidence in this age range in research data on hundreds of children from different cultures, languages, and socioeconomic circumstances. Therefore, it can be concluded that a vast majority of children master this skill, which has been considered central in the development of ToM, at the beginning of primary education.

Beyond the ability to attribute false beliefs, there is considerable evidence that progress in the development of ToM continues to be made during the years of primary education [18,19,20]. After age six, this development is characterized by an increase in the ability to simultaneously manage a variety of mental states in complex social settings; this has been evidenced by the development of new tasks, and even batteries, which allow the study of the evolutionary advances in ToM. Thus, the construct was initially extended with the tasks of attribution of false belief of the second order [21], the interpretation of non-literal meanings such as irony [22], and the understanding of mistakes [23]. The changes shown in studies assessing this advanced ToM appear to be associated with age-related improvements in verbal skills, but primarily the cognitive skills of children in mid-childhood.

The relationship between ToM and language ability has been widely documented, although it mainly refers to early ToM developments. Language is a fundamental tool for the development of the communicative interactions that stimulate the development of ToM; conversations with adults and peers expose children to the different perspectives that people possess [24]. Therefore, there is an indirect link between ToM and language ability. In addition, the representation and expression of mental states require a “mentalist” vocabulary, i.e., a vocabulary that allows us to put into words meanings such as knowing, believing, thinking, assuming, etc., that do not refer to directly observable behaviors [25]; likewise, grammatical competence allows children to reason about the contents of other minds and to represent relationships between mental states such as those expressed in the grammatical structure “he doesn’t know that she knows”, typical of the advanced ToM [26].

Regarding the relationship between ToM and cognitive development, Carlson et al. [27] exposed several reasons that justified the relationship between Executive Function (EF) and ToM during development, referencing reasons such as: self-control and understanding of false belief develop simultaneously in time; ToM and EF share brain areas such as the frontal lobe, which implies that an alteration in this area can alter both developments; both are affected in neurodevelopmental disorders such as autism spectrum disorder (ASD); and performance in some ToM tasks requires behavioral inhibition and working memory. Studies on the effect of bilingualism on ToM performance, specifically in participants with ASD and typical development (TD), also provide evidence of the role of behavioral inhibition in ToM. The study of Baldimtsi, Peristeri, Tsimpli, and Durrleman (2020) [28] shows a significant positive effect of bilingualism in both TD and ASD groups. Their findings support the hypothesis that bilinguals’ ability to constantly inhibit one of their languages during communication may contribute to a generally enhanced inhibitory control system. This may consequently result in more effective inhibition of one’s personal knowledge during a ToM task. Closely related to the previous study, Peristeri, Baldimtsi, Andreou, and Tsimpli’s (2020) [29] findings reveal that bilingual children with ASD outperformed their monolingual peers with ASD working memory skills.

In the early childhood education stage, there is evidence of links between different measures in EF and the ability to attribute false beliefs [30]. However, EF is a very broad cognitive construct that integrates various components. In later childhood and adolescence, the overall cohesion between these skills changes [31,32], evolving in a non-unitary way, which leads to the need to consider the components of EFs separately when researching older children. Working memory (WM) is a fundamental component of EF, involving the ability to consider and manipulate different types of information over a short period of time. The correlation between WM and ToM during primary education has been shown in recent studies [33,34], finding data that support ToM as one of the mechanisms underlying the development of WM at these ages [35,36]. Specifically, in the last of the cited studies, after applying a program based on conversations about ToM, it is shown that individual differences in WM skills predict the possibility of children taking advantage of the program and therefore, the development of their ToM skills. The most relevant conclusion from the mentioned studies is that WM is related to ToM not only because the tasks that serve to assess ToM demand the use of WM by the individuals assessed, but also, and more importantly, because the skills involved in WM allow children to take advantage of social experiences that are relevant to improving their understanding of the minds of others. Being able to keep in mind and process different information allows children to consider and integrate different perspectives of the same event, and this makes it possible to generate a coherent vision of it.

The study by Im-Bolter et al. [37] integrates both cognitive and linguistic aspects in explaining the development of ToM. Their results show that both EF and language play an important role in the performance of ToM in children between 7 and 8 years of age and in pre-adolescents (11 and 12 year olds). The authors explain that EF and language contribute differently to the development of ToM, with EF allowing individuals to compare and contrast different points of view to determine and evaluate the intention behind the behavior, while the semantics of language provides the vehicle for the representation of increasingly sophisticated intentions, beliefs, and emotions.

### 1.2. Cognitive Profile of ADHD

In recent years, interest has begun to emerge in the scientific community to develop cognitive profiles that help both to better understand the disorder and to evaluate and treat it [4,38,39,40]. One of the standardized tests used for this purpose has been the Wechsler Intelligence Scale for Children—Fourth Edition (WISC-IV). Various studies indicate that people with ADHD do not differ from healthy controls in their total intellectual capacity. The difference is found in that people with ADHD show higher scores on the General Capability Index (GCI)—understood as the sum of the scores obtained in Verbal Comprehension (VC) and Perceptual Reasoning (PR)—than on the Cognitive Capability Index (CCI)—understood as the sum of WM and Processing Speed (PS). In other words, the scores on the WM and PS scales are worse than those obtained in VC and PR [4,40,41,42]. Navarro-Soria et al. [4] specify that relevance lies not in a low score in WM or PS, but in a lower score on these indices in comparison to others obtained by the subject. 

As far as WM is concerned, people with ADHD present less efficiency in the codification of the stimuli and a marked tendency to “prolong” the activation of the current stimulus. For this reason, they are classified as “forgetful” and as “only living in the present” without paying attention neither to the future consequences of their actions nor to the projection towards goals in time. However, this is mainly due to a less adequate use of coding and remembrance strategies than a structural problem of WM. Therefore, what varies with respect to subjects who do not suffer from ADHD is how they “use” WM [3]. With regard to the PS, there are differences depending on the symptomatology of ADHD. If the symptomatology of hyperactivity and impulsivity prevails, individuals are very fast at processing information and executing the task, but the result of it is negative, (they fail, skip exercises, respond without analyzing all the options, etc.). However, if what prevails is the symptom of inattention, their PS is very low, although the little they do is with a positive result.

### 1.3. Relationships between Attention Deficit Hyperactivity Disorder, Theory of Mind, and Working Memory

The proposal of a deficit in ToM as a secondary symptom associated with ADHD, as a result of a deregulation of the executive system, is due to research that refers to an ideal inhibitory control and functioning of the working memory for an adequate Theory of Mind [43]. This hypothesis would be coherent with the idea [2] of a central deficit of behavioral inhibition in ADHD that would alter the correct functioning of the EFs. In line with this, Lavigne and Romero [3] differentiate between the Executive System (in charge of directing and coordinating the actions of the body) and EFs (capacities that allow, voluntarily, the regulation of actions of the body); the primary deficit goes beyond the alteration of behavioral inhibition as a central deficit, because there is a deregulation of the Executive System that affects the poor performance of the Executive Functions. Consequently, an alteration at the executive level makes it difficult to anticipate and to infer, altering other elements belonging to social cognition, among them the ToM.

Research, such as that of Huang-Pollock et al. [44], showed that the alteration of executive functions in ADHD could cause a worse acquisition and deficient use of social skills in children from 8 to 12 years old. Specifically, they found problems in the detection of verbal social cues and the memory or follow-up of the conversation, alluding to the fact that these results were due more to the characteristic inattention of the disorder, than to impulsivity and hyperactivity.

In a meta-analysis of studies conducted between 1979 and 2009, Uekermann et al. [45] pointed out that although data are scattered, there is evidence of deficits in ToM in individuals with ADHD, compared to controls, especially when ToM should be applied in everyday situations. 

The study conducted by Caillies et al. [46] measured performance on second-order false belief tasks, non-literal meaning, and executive functions in two groups of participants (all 9 years old), a control group and a group with ADHD. Their data showed significant differences in performance in second-order tasks, understanding of ironies, and executive functions between the control and experimental groups, with lower scores in the experimental group.

Bora et al. [47] carried out a meta-analysis studying performance in social cognition tasks (emotion recognition and ToM) in groups of people diagnosed with ADHD, ASD, and control groups. In their analysis, they found that performance in these variables was lower in the groups diagnosed with ADHD compared to the control groups; furthermore, the differences between patients with ASD and ADHD were also significant, with the former showing more problems in the mentioned activities and the latter group showing more heterogeneous results. However, when the focus is on second-order false belief attribution tasks, other research found similar performance when comparing groups with ADHD and ASD [13,48,49]. This suggests that difficulties in certain ToM skills may be as pronounced in those diagnosed with ADHD as those of subjects diagnosed with ASD.

After all the above mentioned, it is to be expected that many children with ADHD will not perform at the same level of ToM tasks as typically developing children. In addition, as noted before, previous studies have shown that WM and VC are predictors of the development of ToM in typically developing children. Therefore, since children with ADHD are characterized by low levels of development in WM but not in VC, it could be expected that WM would be the factor to best explain the inter-individual differences in ToM. However, given the role of language in the development of ToM, it was also thought that the participants’ VC would help facilitate performance in ToM by modulating the effect of WM. 

Therefore, the general aim of this study is to investigate the relationships between ToM, WM, and VC in primary education students diagnosed with ADHD. To do so, we examined the performance in ToM of children with ADHD between 6 and 12 years old in order to study if their level of development was different from the typical development in that age range. In addition, it was studied whether WM and VC contributed to predicting the performance of these children in terms of their ADHD.

The following specific objectives were set:

1. To examine performance in a wide range of skills linked to the ToM in primary education students with ADHD in order to check if they are in line with typical development.
2. To study whether difficulties in ToM persist in the age range covered by primary education.
3. To examine if WM and VC correlate with the performance of the participants in terms of their ToM.
4. To study the effect of each variable (age, WM, and VC) on the participants’ performance in terms of ToM.

## 2. Materials and Methods

### 2.1. Participants

This study was conducted with a group of 44 primary school students between the ages of 6 and 12. Of these, 72.7% (*N* = 32) were diagnosed with the combined type of ADHD, and 27.3% (*N* = 12) were diagnosed with ADHD with predominantly attention deficit, following the criteria of the DSM-IV-TR [50], or DSM-5 [1] (depending on the moment at which the assessment was carried out, we would use one or the other).

The selection of the subjects took place in collaboration with members of a team from a specific child and adolescent neuroscience center. The principal investigator provided details of the project to the Neuroscience Centre ethics committee, which assisted in the selection of participants and obtained their approval. This study was carried out in accordance with the recommendations of the Faculty of Educational Sciences and the Regulations of the Ethical Committee of Experimentation of the University of Malaga. In addition, it complies with the requirements of the Organic Law on Data Protection 3/2018, in force in Spain. Ethics approval was not required as per the University of Malaga’s guidelines and national regulations.

The following inclusion criteria were used:

- Diagnosis of ADHD.
- Having manifested symptoms for more than 6 months in at least two environments (school, family, and/or social).
- Presence of a significant deterioration of social and/or emotional activity.
- Absence of diagnosis of schizophrenia, psychotic disorder, or any other neurodevelopmental disorder, intellectual disability, and/or sensory or motor deficits.
- IQ equal or greater than 80.
- Primary school students
- Not having received—nor currently receiving—specific treatment in the field of social cognition.

Secondly, contact was made with the families, informing them about the study and presenting them with the “Informed Consent” document, where the justification of the study, the objectives, and the procedure to be followed were mentioned. Subjects who met the criteria and accepted the conditions of the study were included. 

### 2.2. Design

A descriptive and correlational design was chosen, seeking to obtain a causal relationship between several variables: performance in the Theory of Mind task and performance in cognitive tasks, specifically WM and VC. The selection of the sample was non-probabilistic, due to chance not being used. Only those participants who met specific inclusion criteria were admitted to the study.

### 2.3. Instruments

All participants were evaluated using the following two instruments:

The NEPSY-II Neuropsychological Battery [51]. From this battery, the Theory of the Verbal Mind (TVM) subtest corresponding to the social perception domain was used. The age of application is from 3 to 16 years old and evaluates a series of mental functions directly related to ToM [51]. This test consists of 15 items in total which, according to its authors, evaluate the ability to understand mental functions such as belief, intention, deception, emotion, imagination, and pretense, as well as the ability to understand that others have mental states which may be different from one’s own. A more exhaustive analysis of the subtest leads us to point out that nine of its items measure a series of abilities typical of ToM (attribution of beliefs, first-order false belief, deception, double deception, and non-literal language), while six of them assess ToM prerequisites such as imitation, the distinction between mental and physical, and the differentiation between appearance and reality.

The TVM subtest requires the participation of both comprehension and verbal expression skills, even when supported by images. The procedure in each item is similar: the individual being tested is shown a picture, the situation related to the picture is described, and then, a question is asked that requires the individual to place him/herself in another person’s point of view in order to be answered correctly. The instrument provides a scale of percentiles, which allows the scores obtained by the individual assessed to be placed in relation to those of individuals with typical development of their age.

Wechsler Intelligence Scale for Children IV or WISC-IV [52]. The age of application is from 6 years to 16 years and 11 months. This neuropsychological test measures different cognitive abilities. In this study, the data referring to the scores of the WM and VC indices are provided. The WM index is obtained by adding the scores from the digits and letters and numbers subtests, while the VC index is obtained by adding the scores from the vocabulary, similarity, and comprehension subtests. These scores allow for the comparing of participants to individuals with typical development for their age.

According to the authors of the test, the assessment of WM provides data on the subjects’ ability to temporarily retain certain information in memory, work or operate with said information, and generate a result; it involves attention, concentration, mental control, and reasoning. Regarding the assessment of VC, it informs about the capacity to define and establish semantic relationships between concepts, as well as to answer a series of questions about social situations or general principles. 

### 2.4. Procedure

With the collaboration of specialists in the field of psychology, speech therapy, education, and neurology and team members of a specific Centre for Child and Adolescent Neuroscience in Malaga, we proceeded to contact, by telephone, the families of children diagnosed with ADHD who met the inclusion criteria, informing them of the study and requesting their voluntary participation. Later, those interested were called for an interview where the research was explained in detail and the interested parties signed the informed consent document.

For the evaluation of ToM, WM, and VC, two sessions of approximately one and a half hours each were used, in which the participants were evaluated individually. In the first session, the subtests that make up the WM and VC were passed, while the ToM tasks took place in the second session. 

The time schedule of our experiment (recruitment of the sample and subsequent evaluation) lasted four months.

### 2.5. Data Analysis

Participants were placed into three groups according to their centile scores in ToM (group 1 from 0 to 25; group 2 from 26 to 50; group 3 from 50 onwards), and the frequency and percentage of participants for each group were obtained. Likewise, the means and standard deviations of the WM and VC indices of each group were calculated. Pearson’s correlation analyses were carried out to analyze whether there was a relationship between the age of the participants and the centile score obtained in ToM, as well as to study whether there was a relationship between ToM, WM, and VC. Finally, a multiple linear regression analysis was performed with the enter method to check the effect of WM and VC on ToM.

## 3. Results

Table 1 presents the descriptive data on the distribution of participants in relation to the centile score achieved in ToM, as well as the means and standard deviations of WM, VC, and chronological age for each group.

As can be seen in Table 1, there is a high percentage of participants who score below the typical development in ToM. In addition, it is noteworthy that the WM scores are lower than the VC scores. 

In order to check whether difficulties in ToM persist throughout the age range covered by primary education, an analysis of correlation between age and performance in ToM tasks was carried out. The results indicated that the correlation between both variables was not statistically significant (−0.081, *p* = 0.600). It is worthy to note the negative sign of the value of Pearson’s correlation coefficient, since it indicates that although there is no difference in the centile score between the younger and older participants, if there were, it would be in the negative direction—that is, the older participants would have the worst centile score.

The relationships between ToM, WM, and VC were also analyzed. The results showed that the relationship between WM and ToM was statistically significant (0.511, *p* = 0.000) as well as between VC and ToM (0.389, *p* = 0.012). In addition, WM and VC significantly correlated with each other (0.337, *p* = 0.031). Since a correlation between WM and VC was found, a partial correlation analysis between WM and ToM was performed, controlling for the effect of VC, in order to ensure that the relationship between WM and ToM was not influenced by the effect of VC on WM. The results show that, once the effect of VC on WM was controlled, the relationship between WM and ToM was still statistically significant (0.408, *p* = 0.009).

**Figure 1:**
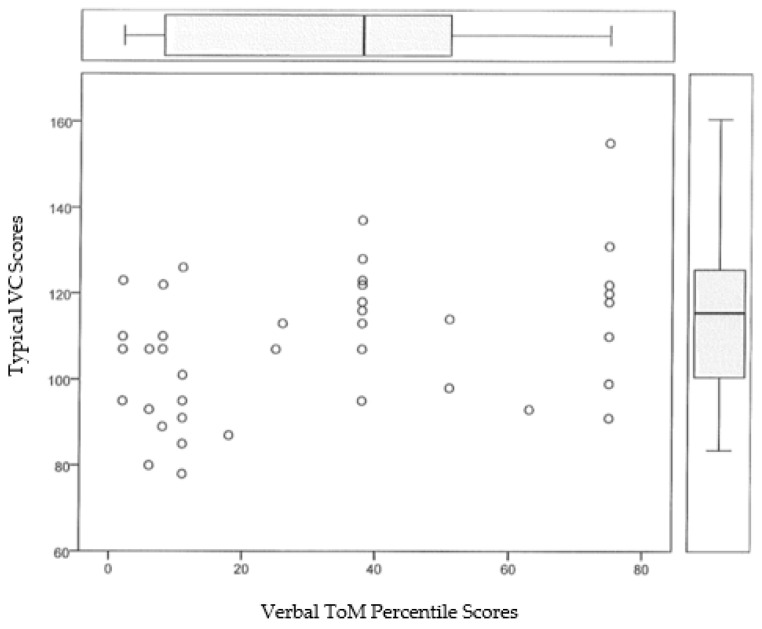
Representation of the effect of the WM on ToM in the model.

Finally, taking as dependent variables (DVs) the centile score obtained in ToM and as independent variables (IVs) the scores in WM and VC, a multiple linear regression analysis was performed with the enter method. The assumptions of linearity, independence, homoscedasticity, normality, and non-collinearity have been fulfilled. The data are shown in Table 2 and Figure 1.

The statistical data show that of the two independent variables, WM is the significant variable and that the model explains 29.4% of the variance obtained in the data referring to the centile score in ToM by the participants.

## 4. Discussion

This study has examined performance in a wide range of skills linked to ToM in primary school students with ADHD, as well as its relationship to their level of development in VC and WM.

In regard to the first objective, the results show that a high percentage of students perform below the expected typical development range, placing them in very low percentiles. These findings are in line with those found by Caillies et al. [46] and Uekermann et al. [45]. Da Fonseca et al. [53] compare the performance of two groups of children and adolescents (one diagnosed with ADHD, and the other without ADHD) in basic ToM tasks: recognition of emotions through faces and taking into account contextual information. Significant differences are found to the detriment of the ADHD group for both types of tasks, where the subjects diagnosed with the disorder are less accurate when making inferences about the emotional states of others. Unlike other studies [54], in which data on development in ToM come from information provided by the parents and teachers of students with ADHD, this study used a battery of ToM tests that directly assess participants. Although it is interesting to know whether the difficulties in ToM have an effect on everyday situations, as shown in these works, it is also important to be able to verify that these difficulties in students with ADHD are greater than those of their peers, with this only being possible if standardized assessment tests, such as those of this study, are used.

Showing these difficulties is relevant, since this characteristic for many people with ADHD only started drawing attention a few decades ago. For many professionals in education and psychology, this is not a feature to highlight in the profile of a student with ADHD, unlike other neurodevelopmental disorders (such as ASD) in which this difficulty is part of their profile. As we pointed out in the introduction, when the focus is placed on second-order false belief attribution tasks, we found research that observes a similar performance when comparing groups with ADHD and ASD [13,48,49]. This suggests that the difficulties in certain ToM skills of individuals with ADHD may be as pronounced as those of individuals diagnosed with ASD. In contrast, González-Gadea et al. [55] compare executive functioning profiles and ToM in adults diagnosed with ADHD and high functioning ASD, finding a lower performance in tasks involving WM in the ADHD group, while the group of patients with high functioning ASD showed greater social cognition difficulties in ToM, revealing a heterogeneous profile that seems to be unique in subjects with ASD. However, the authors point to a high variability of results (above and below the mean) for the group of ADHD subjects in tasks that involve “Faux Pas”, and suggest that this task is complex and requires a high load attention and WM, which could explain such fluctuations in the results found. The causes that underlie these difficulties in ToM are logically different in diverse disorders, which is why it is important to analyze which variables have explanatory value; in this study, we have also focused on this analysis.

In addition to verifying the existence of these difficulties, this study sought to investigate whether difficulties in ToM persist in the age range covered by primary education (second objective). After analyzing the results, we observed that there is no difference in the centile score between the younger and older participants. Considering the scores obtained by the older and younger participants, it is noteworthy that it is the older participants who are at a lower centile. This leads us to interpret that if, as we pointed out at the start, the improvement in cognitive skills leads to advances in the development of ToM, in the case of participants with ADHD who do not improve as expected in certain cognitive skills, these advances do not occur.

The third aim of this study was to evaluate if WM and the VC correlate with the participants’ performance in ToM. As expected, based on the research on typical and atypical development of ToM cited in the introduction, the relationship between ToM, VC, and WM performance is positive. There is sufficient data to support that putting ToM into function requires representational skills mediated by language, as well as cognitive skills such as the ability to compare and contrast different points of view (one of the functions of WM).

Finally, the fourth objective of this study was to test the independent effect that age, WM, and VC have on participants’ performance in terms of ToM. Analysis of the effect of these variables showed that only WM explained the variance in participants’ performance in terms of ToM. Precisely, as noted above, a lower performance in WM compared to VC is one of the features that characterize the cognitive profile of ADHD [4,41]. The incidence of WM in the development of ToM may be higher in more advanced stages of childhood. In the primary education stage, the development of ToM is more advanced, requiring the cognitive ability to simultaneously manage a variety of mental states, which implies that there must be optimal development in WM. The data of this study do not point to a deficiency in WM; however, the participants do show lower scores in WM than in VC, which leads us to think that WM is not functional enough to successfully face ToM tasks. Sodian and Hülsen [56] found significant differences between ADHD and controls in advanced false belief tasks (where WM would have a central role, due to the amount of information that the subject must keep active during their performance), while the performance between groups was similar in strange stories (which require a greater verbal load, specifically, semantic, and syntactic aspects). In contrast to such results, Charman, Carrol, and Sturge [57] do show significant differences between ADHD and controls in the strange stories task. 

Several studies, such as those by Bora et al. [47], Hutchins et al. [54], Mary et al. [58], and Mohammadzadeh et al. [59], suggest the need for intervention in mentalist skills in the child population diagnosed with ADHD. They consider that with specific intervention, their functioning and behavior could be improved, achieving a greater adaptation to the environment. Mary et al. [58] emphasize the long-term consequences that difficulties in ADHD can have on the social, family, and educational adjustment of people with ADHD.

Conventional programs for the treatment of ADHD usually take into account variables that cause greater problems in social, school, and family adaptation, attributing a leading role to EFs. However, there are few programs that include ToM activities for this purpose. From the results found in the present study and those found by other researchers [5,54], we propose the inclusion of the stimulation of ToM as a tool to improve these functions. Intervention activities in ToM require skills that stimulate WM, such as attending to different points of view (even opposing ones), considering relevant aspects of context, making decisions, inferring mental states, and predicting behaviors. However, since the intervention is usually aimed at primary school children, it would be necessary to focus on advanced skills such as those related to the attribution of first- and second-order false beliefs, understanding metaphors, blunders, ironies, jokes, and making moral judgments. To this end, a series of topics and resources are proposed in Table 3, which can help professionals in the fields of education, psychology, and neurology to develop their work sessions.

## 5. Conclusions

In summary, WM was the only variable that explained the variance in participants’ performance in terms of ToM. In the primary education stage, the development of ToM is more advanced, requiring the cognitive ability to simultaneously manage a variety of mental states, which implies that there must be optimal development in WM. The data of this study do not point to a deficiency in WM; however, the participants do show lower scores in WM than in VC, which leads us to think that WM is not functional enough to successfully face ToM tasks.

It is necessary to incorporate a block of contextualized ToM tasks within the psychoeducational intervention packages for elementary students with ADHD in order to contribute to the improvement of WM. These tasks would stimulate the following processes: (i) addressing the relevant keys to the situation, (ii) interpreting them, and (iii) building a response adjusted to the situation.

Therefore, it would be expected that after the specific intervention, there would be an improvement both in ToM and in WM, thus reducing the problems of adaptation to all the environments where these skills are put into play.

It is important to point out that the results obtained must be considered with caution because one of the main limitations of the present study is the small sample. The homogeneity of the sample has been prioritized (so that only elementary school students with ADHD and without other associated deficits were included). However, it must be recognized that the limited number of participants has been able to condition the significance of the findings and their power to convincingly answering the study objectives might not be high.

All of this would justify that as future lines of research, the possibilities of expanding the study sample are considered. This would allow it to be distributed in different experimental groups in which the differentiated effect of WM on the ToM of the subjects could be verified according to the type of diagnosis (ADHD with a predominance of inattention, ADHD with a predominance of hyperactivity and impulsivity, and combined ADHD), and even compare the results with a normotypic control group.

## Figures and Tables

**Table 1:** Descriptive data on the distribution of participants in relation to the centile score achieved in Theory of Mind (ToM), and the means and standard deviations of working memory (WM), verbal comprehension (VC), and chronological age.

Centile Score ToM	N/%	WM Means (SD)	VC Means (SD)	Age Means (SD)
0–25	22	78.09	100.68	9.5
	50%	(17.97)	(14.25)	(1.84)
26–50	10	94.30	117.20	9.7
	22.70%	(11.00)	(11.54)	(1.49)
51–100	12	99.25	114.25	9.08
	27.3%	(13.28)	(18.06)	(1.97)
0–100	44	87.55	108.68	9.43
	100%	(17.96)	(16.39)	(1.78)

**Table 2:** Predictive validity of independent variables on dependent variables.

Predictors	B	Error	Beta	*t*	*p*
**Constants**	−64.735	26.855		−2.411	0.021
**WM**	0.595	0.216	0.399	2.755	0.009
**VC**	0.420	0.240	0.254	1.754	0.088
**Age**	−2.892	2.026	−0.191	−1.427	0.161
***R***			0.586		
***Square R***			0.343		
***Adjusted Square R***			0.294		

**Table 3:** Proposed sequencing of topics and resources.

Topic Sequencing Proposal	Proposed Resources for Topics
Attend, retain, and interpret the points of view of others, analyzing situations, and foreseeing consequences Attribution of false belief of the first and second order. Discovering and understanding absurdities, deceptions, mistakes, and blunders. Distinguish between deception and error. Analyze jokes and riddles. Make jokes to peers and/or teachers. Interpreting non-literal meanings such as irony and white lies. Making moral judgments. Watching videos that help describe and analyze situations.	In mind 1 [60]. In mind 2 [61]. In mind 3 [62]. In mind 4 [63]. Logokit 2 [64]. Intervention through complex theory of mind stories [65].
